# The effect of orbital-lattice coupling on the electrical resistivity of YBaCuFeO_5_ investigated by X-ray absorption

**DOI:** 10.1038/s41598-019-54772-0

**Published:** 2019-12-09

**Authors:** M. K. Srivastava, X.-S. Qiu, Y. Y. Chin, S. H. Hsieh, Y. C. Shao, Y.-H. Liang, C.-H. Lai, C. H. Du, H. T. Wang, J. W. Chiou, Y. C. Lai, H. M. Tsai, C. W. Pao, H. J. Lin, J. F. Lee, K. Asokan, W. F. Pong

**Affiliations:** 1 Department of Physics, Tamkang University, Tamsui, 251 Taiwan; 2 Department of Physics, National Chung Cheng University, Chiayi, 621 Taiwan; 3 Department of Physics, National Tsinghua University, Hsinchu, 300 Taiwan; 4 Department of Applied Physics, National University of Kaohsiung, Kaohsiung, 811 Taiwan; 5 National Synchrotron Radiation Research Center, Hsinchu, 300 Taiwan; 6 Department of Physics, Banasthali Vidyapith, Rajasthan, 304022 India; 7 Inter-University Accelerator Center, Aruna Asaf Ali Marg, New Delhi, 110 067 India

**Keywords:** Materials science, Condensed-matter physics

## Abstract

Temperature-dependent X-ray absorption near-edge structures, X-ray linear dichroism (XLD) and extended X-ray absorption fine structure (EXAFS) spectroscopic techniques were used to investigate the valence state, preferred orbital and local atomic structure that significantly affect the electrical and magnetic properties of a single crystal of YBaCuFeO_5_ (YBCFO). An onset of increase of resistivity at ~180 K, followed by a rapid increase at/below 125 K, is observed. An antiferromagnetic (AFM)-like transition is close to the temperature at which the resistivity starts to increase in the *ab*-plane and is also observed with strong anisotropy between the *ab*-plane and the *c*-axis. The XLD spectra at the Fe *L*_3,2_-edge revealed a change in Fe 3*d e*_g_ holes from the preferential ${\bf{3}}{{\boldsymbol{d}}}_{{{\bf{x}}}^{{\bf{2}}}{\boldsymbol{-}}{{\bf{y}}}^{{\bf{2}}}}$ orbital at high temperature (300–150 K) to the ${\bf{3}}{{\boldsymbol{d}}}_{{{\bf{3}}{\bf{z}}}^{{\bf{2}}}{\boldsymbol{-}}{{\bf{r}}}^{{\bf{2}}}}$ orbital at/below 125 K. The analysis of the Fe *K*-edge EXAFS data of YBCFO further revealed an unusual increase in the Debye-Waller factor of the nearest-neighbor Fe-O bond length at/below 125 K, suggesting phonon-softening behavior, resulting in the breaking of lattice symmetry, particularly in the *ab*-plane of Fe-related square pyramids. These findings demonstrate a close correlation between electrical resistivity and coupling of the preferred Fe 3*d* orbital with lattice distortion of a single crystal of YBCFO.

## Introduction

Transition metal (TM) oxides with perovskite structure and general formula ABO_3_ (A = alkaline or rare earth metal, B = TM), and/or layered oxygen-deficient (δ) double perovskites with lower symmetry having general formula AA′B_2_O_6-δ_, or AA′BB′O_6-δ_ (A′ = A or Lanthanides; B′ = same as B or different TM)^1,2^ are well known for their fascinating physical properties such as colossal magneto-resistance, high-T_C_ superconductivity, exhibiting a metal-to-insulator transition, multiferrocity, electrochemical properties and others^3–7^. The mechanisms associated with these properties have attached great interest^8–12^. The physical properties of such compounds have been shown to depend on the size of the cations, their distribution, as well as charge, spin, lattice and orbital degrees of freedom^2,5,11,13–15^. Orbital degeneracy, the valence state of the TM ion and hybridization between TM *d*- and O *p*-states are among the most important factors that determine the interesting physical properties^5,15^

The YBaCuFeO_5+δ_ compound is a member of family of layered oxygen-deficient double perovskites and was grown by Er-Rakho *et al*.^2^ in 1988 just a year after the discovery of the well-known high-temperature superconductivity of YBa_2_Cu_3_O_7-δ_ (YBCO)^4^. YBaCuFeO_5+δ_ is a *p*-type semiconductor^14^, although its crystal structure is close to that of YBCO and has been mistakenly identified as a high-temperature superconductor^16,17^. Furthermore, the magnetic ordering in YBaCuFeO_5_ (YBCFO) is antiferromagnetic (AFM) with a Néel temperature (T_N_) of approximately 440 K^6^. Temperature-dependent magnetic susceptibility of YBCFO has demonstrated an unusual magnetic transition around 230 K that has been claimed in terms of commensurate-to-incommensurate magnetic transition^6,13,18^. YBCFO has also been reported to exhibit multiferroicity at high temperatures (~230 K)^6^. Attempts to manifest this property at temperature close to room-temperature (RT) yield technological applications in industry^6,13^. The local electronic and atomic structures of YBCFO have strong effects on electrical transport behavior. For example, studies of electronic structures in YBCO superconductor have shown that the superconducting properties of cuprates are closely associated with ordered O vacancies and cooperative hybridization between Cu 3*d*- and O 2*p-*states. Additionally, the suppression of breathing mode Cu-O bond stretching vibration may weaken or destroy the superconducting properties of YBCO^12^. The doping of Fe atoms into YBCO favors the substitution at the Cu-2 sites (divalent, lying between Y and Ba planes) over Cu-1 sites (monovalent, lying between two Ba layers in one dimensional chain) of YBCO, resulting in semiconducting behavior^9^. In a manner that depends on its concentration, Fe in YBCO changes the properties by reducing the transition temperature, varying the structure and modifying the oxygen and local magnetic ordering to provide stable Fe sites in the YBCFO^19,20^. Castaner *et al*. measured electrical transport behavior in stoichiometric PrBaFeCuO_5_ compound, which is similar to YBCFO, and found that the conduction in the stoichiometric compound involves variable-range hopping phenomena and the movement of carriers in the Fe/Cu-O_2_ plane^21^. These investigations mentioned above suggest a strong correlation between electrical transport and electronic/atomic structures of TM oxides with the perovskite structure. A recent study by Lee *et al*.^15^ on a single crystal of SrFeO_3-δ_ with a majority tetragonal phase revealed that magnetic and charge-related degrees of freedom are coupled with each other and that its electrical resistivity is associated with the commensurate-to-incommensurate charge ordering (CO) transition (the delocalized Fe^3.5+^ state with fractional valence changes to localized Fe^3+^ and Fe^4+^ states upon the CO transition). More recently, a neutron study by some of the co-authors of the present work demonstrated that a single crystal of YBCFO has strongly anisotropic magnetic properties: two AFM-like transitions occur in the ***ab***-plane and a paramagnetic-like feature along the ***c***-axis. They further identified two antiferromagnetic transitions at T_N1_ ~475 K and T_N2_ ~175 K, revealing the commensurate-to-incommensurate magnetic transition at T_N2_ and the formation of a spiral magnetic structure below T_N2_ in which the magnetic moments lie in the ***ab***-plane with a propagation vector along the ***c***-axis^22^.

To the best of our knowledge, the correlation between temperature-dependent electrical resistivity and the charge/preferred orbital/atomic structure of Fe/Cu sites in a single crystal of YBCFO has rarely been studied. The interesting temperature-dependence of resistivity behavior motivated us to study its electrical resistivity and the involvement of the charge/preferred orbital/atomic structure at Fe/Cu sites in YBCFO. In this study, a strong anisotropy of magnetic susceptibility (χ) as a function of temperature (T) is observed. Electrical resistivity (ρ) also unusually increases as the temperature falls at/below temperature of 125 K. It is important to understand the origin of these unusual physical properties of this compound with spectroscopic techniques and to identify the role of local electronic structures at Fe/Cu sites in YBCFO. X-ray absorption near-edge structure (XANES), X-ray linear dichroism (XLD) and extended X-ray absorption fine structure (EXAFS) techniques were used to investigate the charge (or valence state), preferred orbital and local atomic structures around Fe and Cu sites in YBCFO. Fe and Cu *K*-edge XANES indicated that the valence of Fe^3+^ and Cu^2+^ states remain constant in the ***ab***-plane and along the ***c***-axis of YBCFO at various temperatures. Fe *L*_3,2_-edge XLD spectra revealed that Fe 3*d e*_g_ holes changed from the preferred $3{d}_{{{\rm{x}}}^{2}-{{\rm{y}}}^{2}}$ orbital at high temperature (150–300 K) to the $3{d}_{{3{\rm{z}}}^{2}-{{\rm{r}}}^{2}}$ orbital at/below 125 K, demonstrating that the change in the preferred orbital of Fe 3*d* holes is obviously associated with the unusual increase in resistivity at/below 125 K. Fe *K*-edge EXAFS data further reveal an unusual increase in the Debye-Waller factor (DWF) of the nearest-neighbor (NN) Fe-O bond length in the ***ab***-plane at/below 125 K. These results further suggest phonon-softening behavior in the YBCFO, breaking of the lattice symmetry, especially in the ***ab***-plane of the Fe-related square pyramids, accompanied by a change in the preferred Fe 3*d* orbital, causing an unusual increase in the electrical resistivity and anisotropic magnetic behavior at/below 125 K in a single crystal of YBCFO.

## Results and Discussion

**Figure 1:**
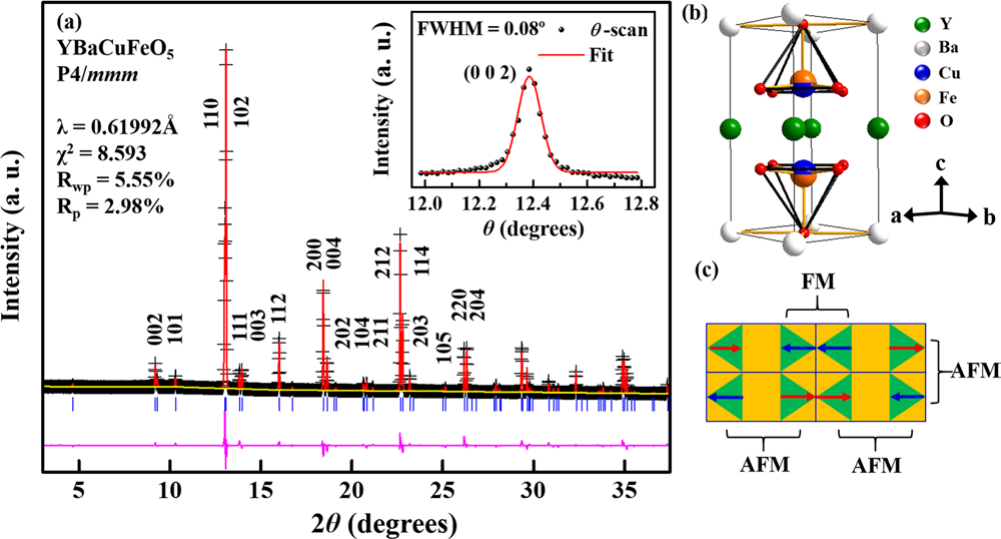
(**a**) Room-temperature X-ray powder diffraction pattern with Rietveld refinement (*P*4*/mmm* space group). Cross marks, a red curve and vertical tick marks indicate the observed pattern, calculated profile and Bragg peaks, respectively. The bottom curve shows the difference between observed and calculated intensities. (**b**,**c**) Three dimensional crystal cell and two dimensional magnetic structures of YBCFO, respectively.

Figure 1(a) displays the X-ray powder diffraction (XRD) of the YBCFO sample at RT. It shows the tetragonal phase with lattice parameters ***a*** = ***b*** = 3.8716 Å and ***c*** = 7.6570 Å, and low-temperature (90 K) XRD (not shown here) also indicates the constant tetragonal phase with lattice parameters, ***a*** = ***b*** = 3.8683 Å and ***c*** = 7.6374 Å of YBCFO, which are consistent with the literature^18^. Rietveld analysis of the XRD pattern was performed by assuming random distribution of Fe and Cu atoms in the Fe/Cu-O_2_ layers, although the literature is inconsistent regarding the positions of Cu and Fe, which by some are considered to form an ordered structure, occupying fixed and distinct sites for those atoms in the lattice (*P*4*mm*, noncentro-symmetric space group)^2,23^, but by others are considered to form a disordered random structure (*P*4*/mmm*, centro-symmetric space group)^24,25^. Therefore, the difference between *P*4*mm* and *P*4*/mmm* is mainly one of symmetry as the structural parameters of YBCFO differ insignificantly^18,22,26^. The structural parameters that were derived by Rietveld analysis are the same as those obtained by the co-authors of this work based on the assumption that a single crystal of YBCFO had the *P*4/*mmm* space group^18,22^. The reliability factors (R_wp_ and R_p_), depicted in Fig. 1(a), reveal close agreement between experimental data and the fitted result. The inset in Fig. 1(a) also displays a Bragg reflection (002) with a small full width at half maximum (FWHM) of approximately 0.08°, indicating that the single crystal of YBCFO at RT was of high quality. Noticeably, the crystal was also treated under different annealing processes, and did not show significant changes in both T_N1_ and T_N2_ as mentioned above. This suggests a stoichiometric composition of YBCFO. Figure 1(b) displays CuO_5_ and FeO_5_ square pyramids that are separated by Y^3+^ planes, with Ba^2+^ ions located at the corners in the tetrahedral lattice, and O atoms at two crystallographic sites O_basal_ and O_apical_ at the basal plane and apical direction of the Fe/Cu pyramids, respectively. Figure 1(c) presents four unit cells that exhibit the magnetic structure of YBCFO that was suggested by Morin *et al*.^13^. The spins, denoted in red and blue, are related to the Fe (or Cu) and Cu (or Fe) cations in Fig. 1(c), respectively. It also suggests that the magnetic coupling between Fe and Cu cations is AFM in the ***ab***-plane, whereas along the ***c***-axis, it alternates between AFM (between cations that do not share O_apical_) and ferromagnetic (FM) in bi-pyramidal blocks that share O_apical_ atom. The crystal structure of YBCFO is generally similar to that of YBCO with respect to the presence of Fe/Cu-O_2_ planes that are separated by Y planes along the ***c***-axis and dissimilar in terms of the Cu-1 sites which form a chain and the BaO layers that are not present in YBCFO^18^.

**Figure 2:**
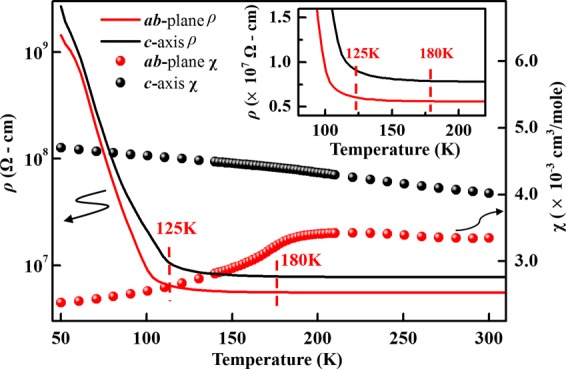
ρ and χ vs. T for single crystal YBCFO measured in the ***ab***-plane and along ***c***-axis. Inset magnifies ρ vs. T curve.

Figure 2 plots the variations of ρ and χ of a single crystal of YBCFO with temperature (T), measured in the ***ab***-plane and along the ***c***-axis. Clearly, Fig. 2 shows strong magnetic anisotropy: χ measured in the ***ab***-plane exhibits an AFM-like transition at approximately 180 K, which is consistent with the literature^13,18,22,24,27–29^ and a paramagnetic-like feature along the ***c***-axis. The AFM-like transition in the ***ab***-plane is reportedly the commensurate-to-incommensurate AFM transition that involves various magnetic unit cells^13,24,27^, whereas anisotropy in the ***ab***-plane and along the ***c***-axis may be associated with the preferential orbital occupancies of the highly directional Fe/Cu 3*d* electrons, whose spins contribute their moments differently in the directions of measurement. Morin *et al*.^13^ presented a model [Fig. 1(c)] concerning of the distributions of Fe and Cu cations in YBCFO and their magnetic structure at 230 K (which is the AFM-like transition temperature, as is approximately 180 K in the present case) that demonstrated the presence of Fe/Cu dimers in the pyramids. They also noted strong AFM coupling in the ***ab***-plane, caused by the super-exchange phenomena, which alternates along with spiral AFM-FM coupling along the ***c***-axis. Therefore, the appearance of the paramagnetic feature along the ***c***-axis can be understood as being caused by weak and unstable coupling between Fe and Cu cations along the ***c***-axis.^13^ Although the temperature dependence of susceptibility do not follow standard 1/T behavior since it suggests the field-induced transitions. Similar behaviour has been observed by the Ruiz-Aragon *et al*.^27^. The coexistence of different phases in the background may be responsible for such characteristics^27–30^. Recently, Dey *et al*.^30^ and Scaramucci *et al*.^31^ addressed theoretically the relative exchange coupling strengths in YBCFO and the role of magnetic exchange interactions and the effect of spin interactions. Additionally, ρ vs. T curves are similar in both directions of measurement in the YBCFO and remain mostly insensitive to temperature during cooling of the sample from RT to ~180 K, but thereafter increases slowly (see the magnified view in the inset of Fig. 2) before exhibiting an unusual rapid increase at/below 125 K. The resistivity with temperature reported here is different from that of Klyndyuk and Chizhova work early^14^. Their study was on polycrystalline compounds synthesized by solid state reaction and possibility of oxygen rich as reported by them. These authors also reported that even variations of 0.05% in cation and anion lead to significant changes in the resistivity since the electrical properties of ferrocuprates depend on the vacancies of the cation and oxygen. Present study is based on the single crystals of YBFCO. Such variation in the electrical resistivity of YBFCO may be associated with the sample preparation and oxygen content.

The origin of the anisotropy in resistivities in the ***ab***-plane and ***c***-axis is understood based on the crystal structure of the tetragonal ferrocuprate YBCFO. This compound is formed by double (Cu, Fe)_2_O_5_ layers of vertex-shared CuO_5_ and FeO_5_ pyramids which are oriented perpendicular to the ***c***-axis. The Ba^2+^ ions are located inside the double layers, and the Y^3+^ ions, between them. The doubling of the perovskite unit cell is the result of the Ba^2+^ and Y^3+^ cation ordering along the ***c***-axis. Thus, the metal–oxygen bonds in the [Cu(Fe)O_2_] plane, as well as the apical oxygen in Cu-O-Fe bonds in the compound YBCFO are different. Such a difference leads to anisotropy in resistivity due to the variation in the Fe(Cu) 3*d*-O 2*p* hybridizations in ***ab***-plane and ***c***-axis. This compound is *p*-type semiconductor. Based on electrical resistivity and thermopower mesurements, Klyndyuks *et al*. concluded that the electrical transport properties of YBCFO can be explained by the small-radius polaron hopping model for these layered ferrocuprates^6^. As the temperature is reduced to 125 K, a large electric polarization sets in due to the magnetism-driven ferroelectricity phase^32^. Kawamura *et al*.^32^ measured the temperature pyrocurrent in YBCFO and observed the pyropeak at ~125 K which is characteristic of the relaxor ferroelectric nature that results in highly resistive regime. These observations can be explained if one assumes that the YBCFO is essentially a combination of the Y_2_Cu_2_O_5_ and BaFeO_3-δ_ phases forming dipoles. However, a similar variation of resistivity is also observed in other perovskites, such as SrFeO_3-δ_ (which includes pyramidal, distorted/tilted, and octahedral Fe sites with various valence states), an effect that is explained in terms of CO and the fact that charge disporpotional^15,33,34^ and the charge density wave-like behavior^35^ occurs at/below the transition temperature. The resistivity ρ in the ***ab***-plane is slightly lower than that along the ***c***-axis. However, the rapid increase in electrical resistivity at/below T~ 125 K in the ***ab***-plane and along the ***c***-axis may arise from the fluctuating valence state, the change in the preferred orbitals of TM 3*d* electrons, and the geometric anisotropies^8^ in the YBCFO. The orbital fluctuation of Mn 3*d* electrons in heavily doped Nd_1-x_Sr_x_MnO_3_ (0.57 ≤ x ≤ 0.75) persists at lower temperatures, giving rise to anomalous ferromagnetic behavior and coexistent high magnetoresistance below the T_N_^36^. The above cited studies reveal that an investigation of the valence state, preferred 3*d* orbital and local lattice symmetry at the Fe/Cu sites would shed light on the rapid increase in electrical resistivity of a single crystal of YBCFO at/below 125 K.

**Figure 3:**
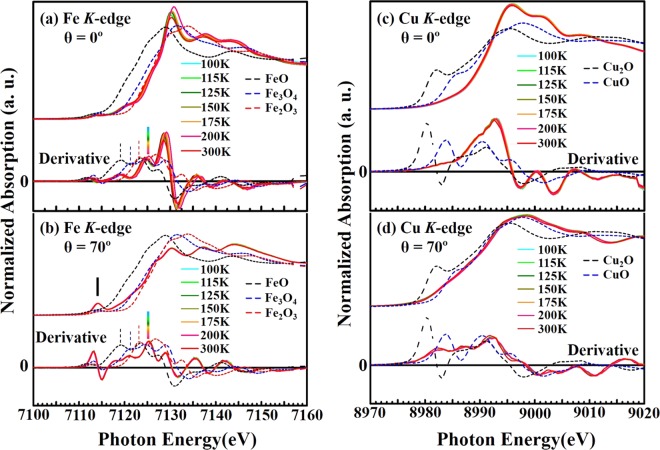
(**a**) Fe *K*-edge XANES spectra of YBCFO recorded at various temperatures for θ = 0° and (**b**) θ = 70°, with corresponding first derivatives (bottom); (**c**) Cu *K*-edge XANES spectra of YBCFO recorded at various temperatures for θ = 0° and (**d**) θ = 70°, with corresponding first derivatives (bottom). XANES spectra of reference samples at room temperature are also presented for comparison.

Figure 3(a–d) display the temperature-dependent Fe and Cu *K*-edge XANES spectra of YBCFO, with the ***E***-field of the incident light parallel to the ***ab***-plane (angle of incidence, θ = 0°) and the ***E***-field nearly parallel to the ***c***-axis (θ = 70°). Compounds of known oxidation state are used for references, FeO (Fe^2+^), Fe_3_O_4_ (Fe^8/3+^) and Fe_2_O_3_ (Fe^3+^) powders at RT are used to determine Fe valence and Cu_2_O (Cu^+^) and CuO (Cu^2+^) powders for Cu valence. All spectra in Fig. 3(a,b) have a common weak pre-edge feature and intense main absorption at the Fe and Cu *K*-edge, whose first derivatives are also shown at the bottom of the figures to reveal the dependence of the rising edge (or threshold) position on two orientations and various temperatures. The Fe/Cu *K*-edge absorption feature is generally governed by the Fe/Cu 1 *s* → 4*p* dipole transition (Δ*l* = ±1) whereas the additional weak transition, called the pre-edge, is governed by the Fe/Cu 1 *s* → 3*d* quadrupole transition (Δ*l* = ±2). The rising edge in the spectrum (first maximum of the derivative) of YBCFO (θ = 0°) (at 7125.3 eV, indicated by the color solid bar) is at a higher energy than that of the reference FeO (Fe^2+^) (at 7119.2 eV, black dashed line) or Fe_3_O_4_ (Fe^8/3+^) (7121.3 eV, blue dashed line), but close to that of Fe_2_O_3_ (Fe^3+^) (7123.0 eV, red dashed line). Notably, the general rising edge or threshold feature of YBCFO is observed highly the superposition with that of Fe_2_O_3_ (Fe^3+^) than that of FeO (Fe^2+^) and Fe_3_O_4_ (Fe^8/3+^). It is well known that trivalent Fe ion usually prefers octahedral or tetrahedral coordination in compound. However, in YBFCO compound, the double layers of square pyramids (Fe/Cu-O_5_) are sharing the apical O with Ba ions, and Y cations are located between the layers. Two types of pyramids FeO_5_ and CuO_5_ are dissimilar in charges and are also with two unrelated symmetry mixed-metal Fe(Cu)O_2_ layers. Furthermore, some studies support the acentric character of this crystal structure. Thus, the valency of Fe appears slightly higher than 3 + that may be associated unique spectral features of Fe ions in the Fe(Cu)O_5_ layers. Thus, there is a variation in the local crystal fields of Fe ions between Fe_2_O_3_ and YBCFO. As mentioned above the metal-oxygen bonds in the [Cu(Fe)O_2_] plane, as well as the apical oxygen in Cu-O-Fe bonds in the compound YBCFO are also different. In Fig. 3(b), the first maximum of the derivative of the rising edge of the Fe *K*-edge of YBCFO for θ = 70° is fairly close to that for θ = 0°. These observations indicate that the mean valence state of Fe in YBCFO is close to that of Fe^3+^. The general spectral feature in Fig. 3(a) is similar to that of PrBaFeCuO_5+δ_ reported elsewhere^21^, in which small features between the pre-edge and the main absorption edge are associated with the pyramidal environment with the Fe^3+^ valence state, apparently owing to the mixing of Fe 4*p* and 3*d* states^37^. The general line-shape and rising edge position of Fe *K*-edge XANES at the orientations θ = 0° and 70° are clearly observed to be insensitive to the measured temperature (100–300 K), exhibiting the stability of the valence around Fe sites of the ***ab***-plane and ***c***-axis.

Likewise, in Fig. 3(c,d), the sharp feature (at the 8980–8985 eV) in the Cu *K*-edge XANES spectra of the reference samples arises from the dipole-allowed Cu 1 *s* → 4*p*(π^*^) non-bonding transitions, which are not observed in YBCFO for θ = 0°. In contrast, this general feature is present in the spectrum of YBCFO for θ = 70°, because the 1 *s* → 4*p*(π^*^) non-bonding transitions occurs perpendicular to the ligand axis, as suggested by Tolentino *et al*.^38^. Therefore, the Cu *K*-edge near-edge absorption in YBCFO is primarily caused by the 1 *s* → 4*p*(σ^*^) anti-bonding (θ = 0°) and 1 *s* → 4*p*(π^*^) non-bonding transition (θ = 70°). The general line-shape and position of the rising edge in Cu *K*-edge XANES are observed to be insensitive to the temperatures (100–300 K), also revealing the stability of the valence around Cu sites in YBCFO. Furthermore, the two orientations obtained from the Cu *K*-edge XANES spectra presented in Fig. 3(c,d) are very similar to those obtained from Cu *K*-edge of La_2_CuO_4_ (Cu^2+^ valence)^38,39^, indicating the primary Cu^2+^ valence state at Cu sites in YBCFO, which is also identified by the superposition of the threshold feature of YBCFO and that of CuO (Cu^2+^). Clearly, based on the results of Fe and Cu *K*-edge XANES spectra, the temperature independence of Fe and Cu valence states does not support the possibility that CO or charge disproportional effects^15,33,34^ are the major responsible for the unusual rapid increase in electric resistivity of the single crystal of YBCFO at/below 125 K.

**Figure 4:**
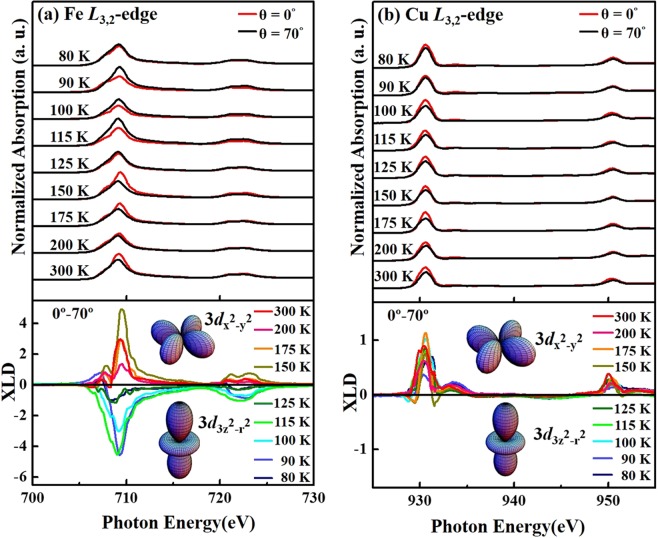
(**a**) Normalized Fe *L*_3,2_-edge XANES spectra (for θ = 0° and 70°) and XLD (bottom panel) of YBCFO. (**b**) Normalized Cu *L*_3,2_-edge XANES spectra (for θ = 0° and 70°) and XLD (bottom panel) of YBCFO.

Figure 4(a,b) show the temperature-dependent Fe and Cu *L*_3,2_-edge XANES and their corresponding XLD (bottom) spectra, obtained when the ***E***-field of incident light is parallel to the ***ab***-plane (θ = 0°) and nearly parallel to the ***c***-axis (θ = 70°) of the single crystal of YBCFO, respectively. The difference between these two X-ray incidence spectra (θ = 0° and 70°) thus obtained is denoted as XLD, which provides insight into the preferentially unoccupied Fe and Cu 3*d* orbitals. The Fe (Cu) *L*_3,2_-edge XANES spectra in Fig. 4(a) [4(b)] include two features- an *L*_3_-edge around 708 (931) eV and an *L*_2_-edge around 722 (951) eV that are separated by spin-orbital splitting. These features are primarily associated with the Fe (Cu) 2*p*→ 3*d* transitions, and depend strongly on the multiplet structures, which are related to the Fe (Cu) 3*d*-3*d* and 2*p*-3*d* Coulomb and exchange interactions, the local crystal field and the hybridization between Fe (Cu) 3*d* and O 2*p* hybridized states^40^. Fe is surrounded by five O atoms forming a pyramid, therefore, the Fe 3*d* levels are split into $3{d}_{{{\rm{x}}}^{2}-{{\rm{y}}}^{2}}$, $3{d}_{{3{\rm{z}}}^{2}-{{\rm{r}}}^{2}}$, *d*_xy_, *d*_yz_, *d*_zx_ and the last two levels are degenerated. Notably, as shown in Fig. 4(a), the intensity of the main absorption feature of the Fe *L*_3,2_-edge for θ = 0° at temperatures from 300 K down to 150 K, exceeds that of the corresponding feature for θ = 70°, whereas the opposite relationship is observed at/below 125 K. In contrast, the intensity of the main absorption feature in the Cu *L*_3,2_-edge when ***E*** is parallel to the ***ab***-plane (θ = 0°) is always larger than that when ***E*** is nearly parallel to the ***c***-axis (θ = 70°) at all measured temperatures, as shown in Fig. 4(b). The changed preferred 3*d* orbital behavior is evident in the Fe *L*_3,2_-edge XLD results that are clearly obtained at various temperatures [bottom panel in Fig. 4(a)], revealing a change in the sign of the feature at/below 125 K. The observed strong linear dichroism of high-spin Fe^3+^ is beyond expectation, because it has a half-filled 3*d* shell. However, the pyramid crystal field as well as the out-of-plane Fe displacement or lattice distortion may result in the 3*d* orbital anisotropy. Moreover, the XLD could also originate from the magnetic interactions, such as collinear magnetic ordering, either ferromagnetic or antiferromagnetic interactions^41–43^. XLD spectra behave non-monotonically with the temperature. Similar behavior is also observed in polarization-dependent O *K*-edge XANES spectra (Figs. S1 and S2 in the Supplementary Materials). Such variations are observed both in ***ab***-plane and ***c***-axis with temperature. All our XANES data are consistently show these variations and help to conclude that the orbital preferences vary with temperature and anisotropic nature. In contrast, the Cu *L*_3,2_-edge XLD spectra in the bottom panel of Fig. 4(b) indicate that the sign of the XLD feature is positive at all measured temperatures, revealing that Cu 3*d e*_g_ holes always occupy the in-plane $3{d}_{{{\rm{x}}}^{2}-{{\rm{y}}}^{2}}$ orbital in YBCFO, even when the temperature is at/lower than 125 K. Notably, in a study of the correlation between temperature-dependent electrical resistivity and the electronic structures around Fe and Cu sites in stoichiometric PrBaFeCuO_5_ and O-rich PrBaFeCuO_5+δ_, Castaner *et al*. concluded that conduction in a stoichiometric compound involves variable-range hopping in which the carriers move in the Fe/Cu-O_2_ plane in the compound^21^. This finding suggests that the conduction mechanism in a stoichiometric compound is dominated by the Fe/Cu $3{d}_{{{\rm{x}}}^{2}-{{\rm{y}}}^{2}}$ orbitals that lie in the ***ab***-plane. Therefore, the rapid increase in resistivity can change the preferential hole occupation from the in-plane Fe $3{d}_{{{\rm{x}}}^{2}-{{\rm{y}}}^{2}}$ orbital at high temperature (150–300 K) to the out-of-plane $3{d}_{{3{\rm{z}}}^{2}-{{\rm{r}}}^{2}}$ orbital at/below 125 K, the latter does not favor electrical conduction in compound, therefore, the electric resistivity of YBCFO is higher at/below 125 K^44^. Calculations of the electronic structures and magnetic properties of ε-Fe_2_O_3_ from first-principles by Yoshikiyo *et al*.^45^ also revealed that the strong hybridization between Fe 3*d*-O 2*p* states induces a non-zero orbital angular momentum of Fe 3*d* states by partial charge transfer from O 2*p* to Fe 3*d*, creating strong magnetic anisotropy via the spin-orbit interaction. The transfer of electrons from O 2*p* to Fe 3*d* states is accompanied by a change in Fe-O bond distances, which depends on the strength of the orbital overlapping, and induces an orbital moment of the Fe 3*d* states, accounting for the electrical transport and anisotropic magnetic properties^46^. The anisotropic Fe 3*d*-O 2*p* hybridization that is caused by the lattice distortion with *off*-centering shifts Fe^3+^ ions at the octahedral sites of multiferroic GdFeO_3_, inducing strong magnetic anisotropy^47^. Clearly, Fe *L*_3,2_-edge XANES and corresponding XLD results, presented in Fig. 4(a), demonstrate a change in Fe 3*d e*_g_ holes from the preferred $3{d}_{{{\rm{x}}}^{2}-{{\rm{y}}}^{2}}$ orbital at high temperature (150–300 K) to the $3{d}_{{3{\rm{z}}}^{2}-{{\rm{r}}}^{2}}$ orbital at/below 125 K, varying the strength of coupling between the Fe-O hybridization and the distortion of the crystal lattice^48–51^. The distortion of the lattice structure can be primarily responsible for lowering of the energy of either the out-of-plane *e*_g_ orbitals or the in-plane *e*_g_ orbitals. In single crystals of Pr_0.5_Ca_1.5_MnO_4_, increasing orthorhombic distortion causes orbital ordering and changes the electronic properties^50^.

**Figure 5:**
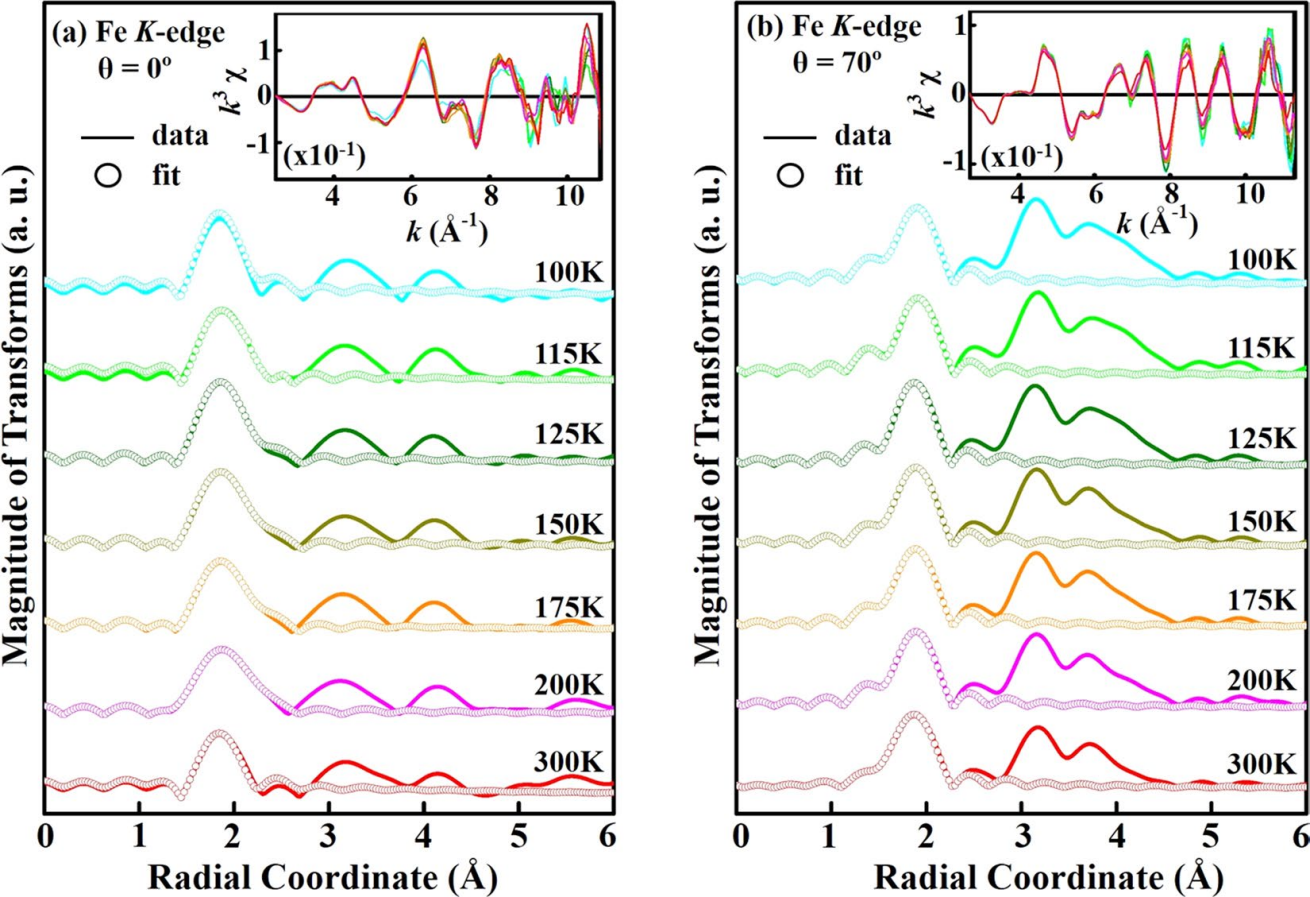
(**a**) Temperature-dependence of amplitudes of Fourier transform of EXAFS at Fe *K*-edge of single crystal of YBCFO for θ = 0° and (**b**) θ = 70°. Inset represents corresponding *k*^3^χ data. Solid profile is obtained from raw data, whereas circular marks represent best fit for first coordination shell.

**Figure 6:**
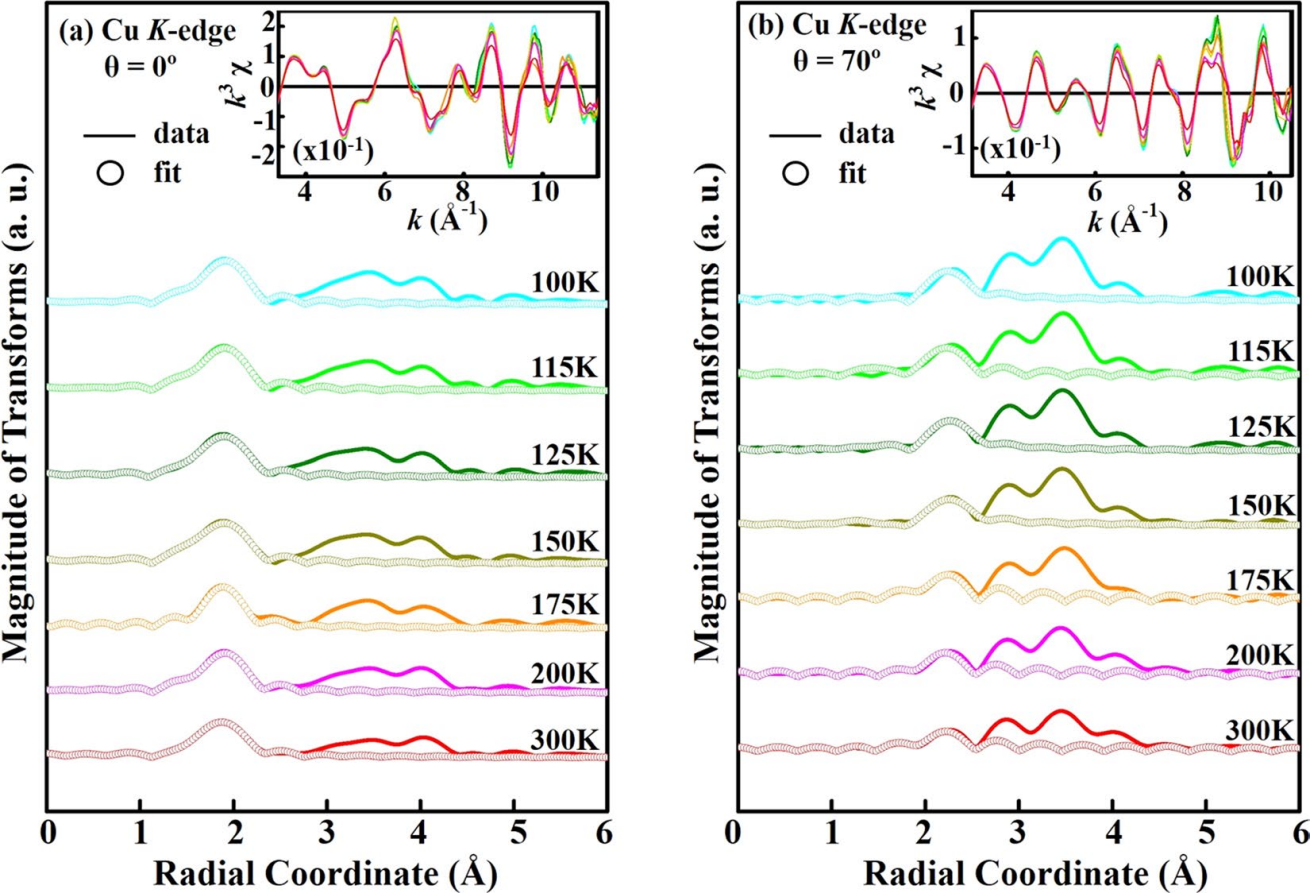
(**a**) Temperature-dependence of amplitudes of Fourier transform of EXAFS at Cu *K*-edge of single crystal of YBCFO for θ = 0° and (**b**) θ = 70°. Inset represents corresponding *k*^3^χ data. Solid profile is obtained from raw data, whereas circular marks represent best fit for first coordination shell.

**Figure 7:**
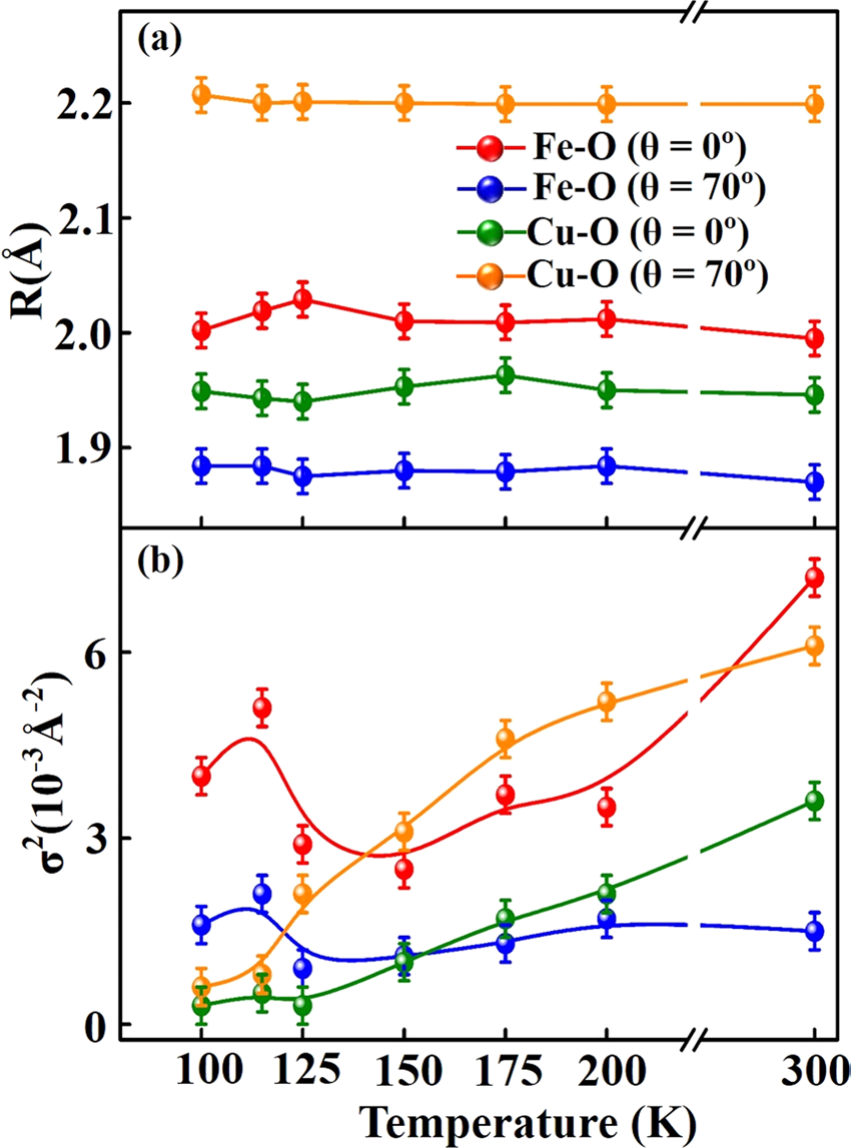
(**a**) Variation of NN Fe/Cu-O bond length in single crystal of YBCFO and (**b**) corresponding DWFs as functions of temperature for θ = 0° and 70°.

To further understand temperature-induced lattice distortion around Fe and Cu sites in YBCFO, the average NN Fe/Cu-O bond length (R), its mean square fluctuation, Debye-Waller factor (DWF) and coordination number (N) are investigated by EXAFS spectroscopy. Figure 5(a,b) show the temperature-dependent magnitude of the Fourier transform (FT) of the Fe *K*-edge EXAFS for θ = 0° and 70° and the fitting of the first coordination shell (NN Fe-O bond length), respectively. The insets show the corresponding *k*^3^ weighted *k*^3^χ oscillating spectra. The selected *k*-range for the fitting (θ = 0° and 70°) was 3.0–10.8 Å^−1^. All spectra were analyzed by standard procedures using the ATHENA program package^52^ to extract quantitative local information (R, DWF and N) about the atomic structure around Fe sites. This work focuses primarily on oxygen coordination around Fe atoms and therefore on the first main feature in the FT spectra of Fe *K*-edge, to examine the variation of the NN Fe-O bond length and corresponding DWF with temperatures, for both polarizations (θ = 0° and 70°). The ultimate results of fitting, presented in Table 1, indicate that the coordination numbers of NN Fe-O for θ = 0° and 70° are 4.0 and 1.2, respectively. Close agreement between the fit and experimental data clearly reveals the square pyramidal environment of O around Fe atoms with a larger basal NN Fe-O bond length than apical distance. The coordination number (N = 1.2) for θ = 70° is fractional because the ***E***-field of the synchrotron photon is not exactly parallel to the ***c***-axis and the ***ab***-plane contributes to the coordination number. The clear pre-edge feature at Fe *K*-edge XANES for θ = 70° [denoted by a solid bar at ~7114.2 eV, Fig. 3(b)] is consistent with the small coordination number along the ***c***-axis, because the intensity of the pre-edge feature is known to increase as the coordination number of Fe complexes decreases, owing to the loss of inversion symmetry at the Fe sites^53^. Fig. 6(a,b) display temperature-dependent FT spectra of the atomic structure around Cu sites for θ = 0° and 70° and fitting results, respectively. The insets show the corresponding *k*^3^ weighted *k*^3^χ oscillating spectra. The selected *k*-range for the fitting (θ = 0° and 70°) was 3.3–11.3 Å^−1^. Like that in the Fe *K*-edge FT spectra, the first main feature in the Cu *K*-edge FT spectra is attributed to the NN Cu-O bond length and it is fitted to determine the structural parameters in Table 2. To show clearly the temperature-induced lattice distortions around Fe and Cu sites in YBCFO, Fig. 7(a,b) display the NN Fe-O and Cu-O bond lengths and their corresponding DWFs at various temperatures, respectively. The NN Fe-O and Cu-O bond lengths in either the basal plane or apical one are almost independent at temperatures. As stated above, Fe *K*-edge FT spectra reveal that in the pyramidal environment, the basal NN Fe-O bond length exceeds than the apical one; in contrast, the apical NN Cu-O bond length is larger than the basal one. Accordingly, the different pyramidal distortions of O are observed around Fe (compressed-like) and Cu (tensile-like) sites, respectively, suggesting that Fe^3+^ (3*d*^5^) and Cu^2+^ (3*d*^9^) ions are located inside the dissimilar symmetrical pyramid. Hence, in a single crystal of YBCFO, Fe^3+^ and Cu^2+^ sites are associated with different lattice distortions or *off*-centering shifts in the ideal pyramidal environment, causing a unique magnetic interaction between Fe^3+^ and Cu^2+^ ions; the magnetic coupling between Fe and Cu ions is AFM in the ***ab***-plane, whereas along the ***c***-axis it alternates between AFM (between Fe and Cu ions) and FM (between Fe and Fe ions) in bi-pyramidal blocks^13^, as presented in Fig. 1(c). Interestingly, as presented in Fig. 7(b), the variation of DWFs at Cu sites typically follows the expected trend, whereas the variation of DWFs at Fe sites deviates from the expected trend at/below 125 K. Generally, the DWF [σ^2^(T)], with two components [σ^2^(T) = σ^2^_stat_+ σ^2^(T)_vib_], varies as exp[−2*k*^2^σ^2^(T)] and is associated with static disorder and thermal vibrations. Component σ^2^_stat_ is related to the static of atomic structure and not related to temperature, whereas σ^2^(T)_vib_ is associated with the lattice vibrations, which typically become smaller as temperature decreases, according to the Einstein or Debye model^54,55^. As expected, at high temperatures (150–300 K), reducing the temperature increases the intensity of the Fe *K*-edge FT feature in the spectrum of YBCFO, because the key factor σ^2^(T)_vib_ is reduced. At/Below 125 K, the intensity of the FT feature decreases markedly as the temperature declines. These anomalous results clearly indicate that σ^2^_stat_ dominates the Fe *K*-edge FT intensity at/below 125 K, revealing that static disorder that are caused by Fe^3+^ ions have a stronger effect than the temperature factor. The large static distortions of the pyramidal oxygen network around Fe sites in YBCFO at/below 125 K, particularly in the ***ab***-plane, can be understood as static distortion contributing highly to the DWF, strongly affecting the FT feature of the NN Fe-O bond. Piamonteze *et al*. observed similar behavior in polycrystalline samples of *R*NiO_3_ (*R* = Pr, Nd, Eu and Y)^56^, which they understood as phonon-assisted behavior at/below the transitional temperature in the compounds. As shown in Fig. 7(b), phonon-assisted behavior at low temperature occurs in YBCFO, especially in the ***ab***-plane, and is related to a reduction or breaking of the crystal lattice symmetry^35,57^. From the analysis of the Fe *K*-edge EXAFS results, the interaction between Fe and O with *off*-centered Fe^3+^ ions in FeO_5_ pyramids in YBCFO is likely to drive strongly phonon softening in the lattice. Typically, soft phonons are associated with phase transitions in crystals that can exhibit more than one lattice symmetry. Thus, the *off*-centered Fe ions or an order-disorder phase transition between dynamic and static distortions, in either process an interaction (that is phonon-mediated) between *off*-centered Fe and O atoms drives phonon softening in the ***ab***-plane of FeO_5_ pyramids. In contrast, as shown in Fig. 7(b), the DWF along the ***c***-axis (at Fe sites) increases slightly as temperature declines at/below 125 K, suggesting competition between thermal and static disorders and certain of phonon-softening behavior. The slope of σ^2^(T) versus T also indicates that Fe/Cu-O_basal_ is more sensitive to temperature than is Fe/Cu-O_axial_ as a larger slope reflects a stronger temperature-dependence. The anomalous variations of DWFs in the ***ab***-plane at Fe sites, which are strongly correlated with the changed Fe *e*_g_ holes from $3{d}_{{{\rm{x}}}^{2}-{{\rm{y}}}^{2}}$ orbital at high temperature (150–300 K) to $3{d}_{{3{\rm{z}}}^{2}-{{\rm{r}}}^{2}}$ orbital at/below 125 K, is believed to be responsible for the electrical resistivity and magnetic properties of YBCFO, as presented in Fig. 2. These results further demonstrate that instabilities in the local NN Fe-O bond length/DWFs and preferred Fe 3*d e*_g_ orbitals drive the metal-to-insulator (or semiconductor) transition in SrFeO_3-δ_^35^ in a manner similar to the driving of the Peierls metal-to-insulator transition in VO_2_, which was elucidated by Budai *et al*. from first-principle calculations^58^. As mentioned above, Dey *et al*.^30^ and Scaramucci *et al*.^31^ investigated theoretically the nature of spiral phase of this compound. Dey *et al*. used the first-principles density functional theory calculation for the YBCFO compound to understand the nature of spiral state based on the role of magnetic exchange interactions and the effect of spin interactions. These calculations indicate that the helical spiral state is more stable at the transition temperature as spins prefers to lie in ***ab***-plane. Scaramucci *et al*. also explored this compound by using Monte Carlo simulations and electronic structure calculations based on density functional theory. By applying the Heisenberg model on a geometrically nonfrustrated lattice with only NN interactions, it was shown that the possibility of spiral phase up to high temperature by a particular type of chemical disorder. They also provided an intuitive explanation to understand this YBCFO. These studies lead to derive a quantitative description of competing orbital, lattice and spin-related degrees of freedom in the YBCFO system.

**Table 1:** Coordination number (N), DWF (σ^2^) and NN Fe-O bond length (R) obtained from fitted temperature-dependent EXAFS spectra at Fe *K*-edge for θ = 0° and 70°.

Temperature	*E*//*ab*-plane (θ = 0°)			*E*//*c*-axis (θ = 70°)		
	N	R (Å)	σ^2^ (Å^−2^) × 10^−3^	N	R (Å)	σ^2^ (Å^−2^) × 10^−3^
300 K	4	2.00 ± 0.02	7.2 ± 0.3	1.2	1.87 ± 0.02	1.5 ± 0.3
200 K	4	2.01 ± 0.02	3.5 ± 0.3	1.2	1.88 ± 0.02	1.7 ± 0.3
175 K	4	2.01 ± 0.02	3.7 ± 0.3	1.2	1.88 ± 0.02	1.3 ± 0.3
150 K	4	2.01 ± 0.02	2.5 ± 0.3	1.2	1.88 ± 0.02	1.1 ± 0.3
125 K	4	2.03 ± 0.02	2.9 ± 0.3	1.2	1.88 ± 0.02	0.9 ± 0.3
115 K	4	2.02 ± 0.02	5.1 ± 0.3	1.2	1.88 ± 0.02	2.1 ± 0.3
100 K	4	2.00 ± 0.02	4.0 ± 0.3	1.2	1.88 ± 0.02	1.6 ± 0.3

**Table 2:** Coordination number (N), DWF (σ^2^) and NN Cu-O bond length (R) obtained from fitted temperature-dependent EXAFS spectra at Cu *K*-edge for θ = 0° and 70°.

Temperature	*E*//*ab*-plane (θ = 0°)			*E*//*c*-axis (θ = 70°)		
	N	R (Å)	σ^2^ (×10^−3^ Å^−2^)	N	R (Å)	σ^2^ (×10^−3^ Å^−2^)
300 K	4	1.95 ± 0.02	3.6 ± 0.3	1.2	2.20 ± 0.02	6.1 ± 0.3
200 K	4	1.95 ± 0.02	2.1 ± 0.3	1.2	2.20 ± 0.02	5.2 ± 0.3
175 K	4	1.96 ± 0.02	1.7 ± 0.3	1.2	2.20 ± 0.02	4.6 ± 0.3
150 K	4	1.95 ± 0.02	1.0 ± 0.3	1.2	2.20 ± 0.02	3.1 ± 0.3
125 K	4	1.94 ± 0.02	0.3 ± 0.3	1.2	2.20 ± 0.02	2.1 ± 0.3
115 K	4	1.94 ± 0.02	0.5 ± 0.3	1.2	2.20 ± 0.02	0.8 ± 0.3
100 K	4	1.95 ± 0.02	0.3 ± 0.3	1.2	2.20 ± 0.02	0.6 ± 0.3

In summary, Fe *K*-edge EXAFS data of YBCFO revealed an unusual increase in the DWFs at/below 125 K in the ***ab***-plane, unlike at Cu sites, where the DWF is typically depend on temperature. This finding suggests that phonon-softening behavior induced lattice distortion in the pyramidal environment around Fe sites and is accompanied by a transfer of Fe *e*_g_ holes from the preferential $3{d}_{{{\rm{x}}}^{2}-{{\rm{y}}}^{2}}$ orbital at high temperature (150–300 K) to the $3{d}_{{3{\rm{z}}}^{2}-{{\rm{r}}}^{2}}$ orbital at/below 125 K, rapidly increasing electrical resistivity and establishing anisotropic magnetic properties in the single crystal of YBCFO.

## Methods

### Synchrotron-based measurements and sample characterizations

Synchrotron-based XRD and temperature-dependent XANES/EXAFS measurements at the Fe and Cu *K*-edge and XANES/XLD measurements at the Fe and Cu *L*_3,2_-edge were carried out at four beamlines (BL-01C2, 17 C, 11 A and 20 A) of the National Synchrotron Radiation Research Center (NSSRC), Hsinchu, Taiwan. Fe and Cu *K*-edge XANES/EXAFS spectra were obtained in fluorescence yield mode, while Fe and Cu *L*_3,2_-edge XANES/XLD spectra were obtained in total electron yield mode. The temperature-dependence of the Fe/Cu *K*- and *L*_3,2_-edge absorption spectra of YBCFO were recorded using two polarizations of X-ray: (i) θ = 0° (***E***-field of linearly polarized photons is parallel to the ***ab***-plane of the YBCFO), and (ii) θ = 70° (the ***E***-field is close to parallel to the ***c***-axis of the YBCFO). Corresponding data were obtained at RT for standard powder samples of FeO, Fe_2_O_3_, Fe_3_O_4_, CuO and Cu_2_O for reference. A single crystal of YBCFO was grown using a modified travelling solvent floating zone technique^18^. ρ was measured as a function of T by the two-point probe method using physical properties measurement system in two perpendicular directions (current parallel and perpendicular to the ***c***-axis of the crystal) as the sample was cooled from 350 to 50 K. The contacts were made of silver paste. χ vs. T was measured using superconducting quantum interference devices. A magnetic field of 1 T was applied along the ***ab***-plane and ***c***-axis and the magnetic anisotropy of the compound was observed.

## Supplementary information

Supplementary information
