# Supplementary material for: The effect of orbital-lattice coupling on the electrical resistivity of YBaCuFeO5 investigated by X-ray absorption
Source: Sci Rep. 2019 Dec 9;9:18586. doi: 10.1038/s41598-019-54772-0 (PMC6901513; doi:10.1038/s41598-019-54772-0)
Supplement: Supplementary file 1 — Supplementary information [file 41598_2019_54772_MOESM1_ESM.doc]

**Supplementary Information**

**The effect of orbital-lattice coupling on the electrical resistivity of YBaCuFeO_5_ investigated by X-ray absorption**

M. K. Srivastava,^1,6^ X.-S. Qiu,^1^ Y. Y. Chin,^2^ S. H. Hsieh,^1^ Y. C. Shao,^1^ Y.-H. Liang,^1^ C.-H. Lai,^1^ C. H. Du,^1^ H. T. Wang,^3^ J. W. Chiou,^4^ Y. C. Lai,^5^ H. M. Tsai,^5^ C.W. Pao,^5^ H. J. Lin,^5^ J. F. Lee,^5^ K. Asokan^7^ and W. F. Pong^1,*^

^1^ Department of Physics, Tamkang University, Tamsui 251, Taiwan

^2^ Department of Physics, National Chung Cheng University, Chiayi 621, Taiwan

^3^ Department of Physics, National Tsinghua University, Hsinchu 300, Taiwan

^4^ Department of Applied Physics, National University of Kaohsiung, Kaohsiung 811, Taiwan

^5^ National Synchrotron Radiation Research Center, Hsinchu 300, Taiwan

^6^ Department of Physics, Banasthali University, Rajasthan 304022, India

^7^ Inter-University Accelerator Center, Aruna Asaf Ali Marg, New Delhi 110 067, India

**S1**: X-ray near-edge structures (XANES) spectra at O *K*-edge of YBCFO corroborate spectroscopic results from Fe(Cu) *K*- and *L*_3,2_-edge to understand the observed temperature-dependent anisotropic magnetic and resistivity.

Fig. S1 shows the normalized O *K*-edge XANES spectra of YBCFO, for θ= 0⁰ and θ= 70⁰, measured at 80, 100, 175 and 300 K to understand the evolution of electronic structures at different transitions. The spectra at θ = 0⁰ and 70⁰ mainly probe the ***ab***-plane and vertical O 2*p* orbitals occupying at basal and apical sites of the Fe/Cu-O_5_ pyramids, respectively. The spectra display four major spectral features, labelled A_1_-D_1_, which are centered at 528.8, 530.6, 533.0 and 536.0 eV respectively. A double and single edge features (A_1_ and B_1_) are evident in the energy region of 528 to 531 eV for θ = 0⁰ and 70⁰ respectively. These features provide detailed information about unoccupied O 2*p*-derived states above Fermi level in YBCFO.^1, 2^ As evident from these spectra, there is significant variation in the spectral features with temperature and non-monotonous. The double-peak feature at the edge is known to originate from the covalent mixing of Fe 3*d* and O 2*p* states, whereas a broad peak-like structures above the threshold is the signature of 4*sp* states of the metal.^1, 2^ The peak, A_1_, at lower energy in double peak structure is related to *t*_2g_-orbital whereas that at higher energy peak, B_1_, is related to *e*_g_-orbital of associated transition metal oxides.^1,2^ The intensity of the *e*_g_-orbital is decreased with increasing number of electrons in *e*­_g_-orbital, e.g., in late TM (Cu) oxides.^1^ By using parallel (θ= 0⁰) and nearly perpendicular (θ = 70⁰) polarization of electric-field vector of synchrotron photon to ***ab***-plan, the information about unoccupied O 2*p*_x,y_-Fe 3*d*_x_^2^_-y_^2^ and O 2*p*_z_-Fe 3*d*_z_^2^_-r_^2^ hybridized states,^3^ respectively, can be extracted. In the case of Cu^2+^, *e*_g_-orbitals are mostly filled and therefore is not prone to strong hybridization with O 2*p* states.^1^ By comparing the O *K*-edge XANES spectra with the standard oxides of Cu and Fe (not shown here), it is observed that all Fe-based oxides (FeO, Fe_2_O_3_ and Fe_3_O_4_) showed double peak feature at the near-edge region as in YBCFO, whereas Cu-based oxides (CuO and Cu_2_O) showed single-peak feature presumably due to the decreased hole occupancy.^1^ Similar double-peak feature in other Fe-based perovskites (LaFeO_3_ and LaFe_3/4_Ni_1/4_O_3_) have also been observed.^4^ The energy range from 526 eV to 532 eV has been integrated after the background (smooth blue dashed line) subtracted at various temperatures in all spectra (θ= 0⁰ and 70⁰) and is plotted in Fig. S2. In a recent study by Suntivich *et al.*,^5^ similar method has been applied to estimate the extent of hybridization of TM 3*d* and O 2*p* states in perovskites. Two different features are observed: (i) the intensity is larger for θ= 0⁰ compared to that of 70⁰ and (ii) the intensity remains nearly temperature insensitive in 300-125 K followed by a gradual decrease, although fluctuations appear around 200-150 K. The spectral features beyond C_1_ to D_1_ and above are due to O 2*p*-Fe(Cu) 4*sp* and Ba(Y) 4*d*(5*d*) states.^2^

The fluctuating behavior may be linked with increased resistivity and unusual susceptibility feature accompanied by commensurate-incommensurate antiferromagnetic (AF) transition that appears in similar temperature range.^6^ Smaller intensity/area for θ= 70⁰ clearly indicates a weak Fe 3*d*-O 2*p* hybridization along ***c***-direction resulting a large resistivity along ***c***-axis as compared to ***ab***-plane.^4^ A relative decrease of hybridization appears at temperatures below 125 K, for both θ= 0⁰ and 70⁰, resulting further enhancement in resistivity. First-principles calculation of electronic structures and magnetic properties of ε-Fe_2_O_3_ (Fe^3+^) by Yoshikiyo *et al.*^7^ reveals that the strong hybridization between Fe 3*d*-O 2*p* states induces a non-zero orbital angular momentum on Fe 3*d* through partial charge transfer from O 2*p* to Fe 3*d* that creates a large magnetic anisotropy through spin-orbit interaction. Moreover, the number of electrons transferring from the O 2*p* to Fe 3*d* accompanied by modification in Fe-O bond distances, depending upon the strength of the orbital overlapping, induces orbital moment of Fe 3*d* differently. This accounts for the anisotropy in magnetic and transport properties as observed by us in Fig. S2.^8,9^


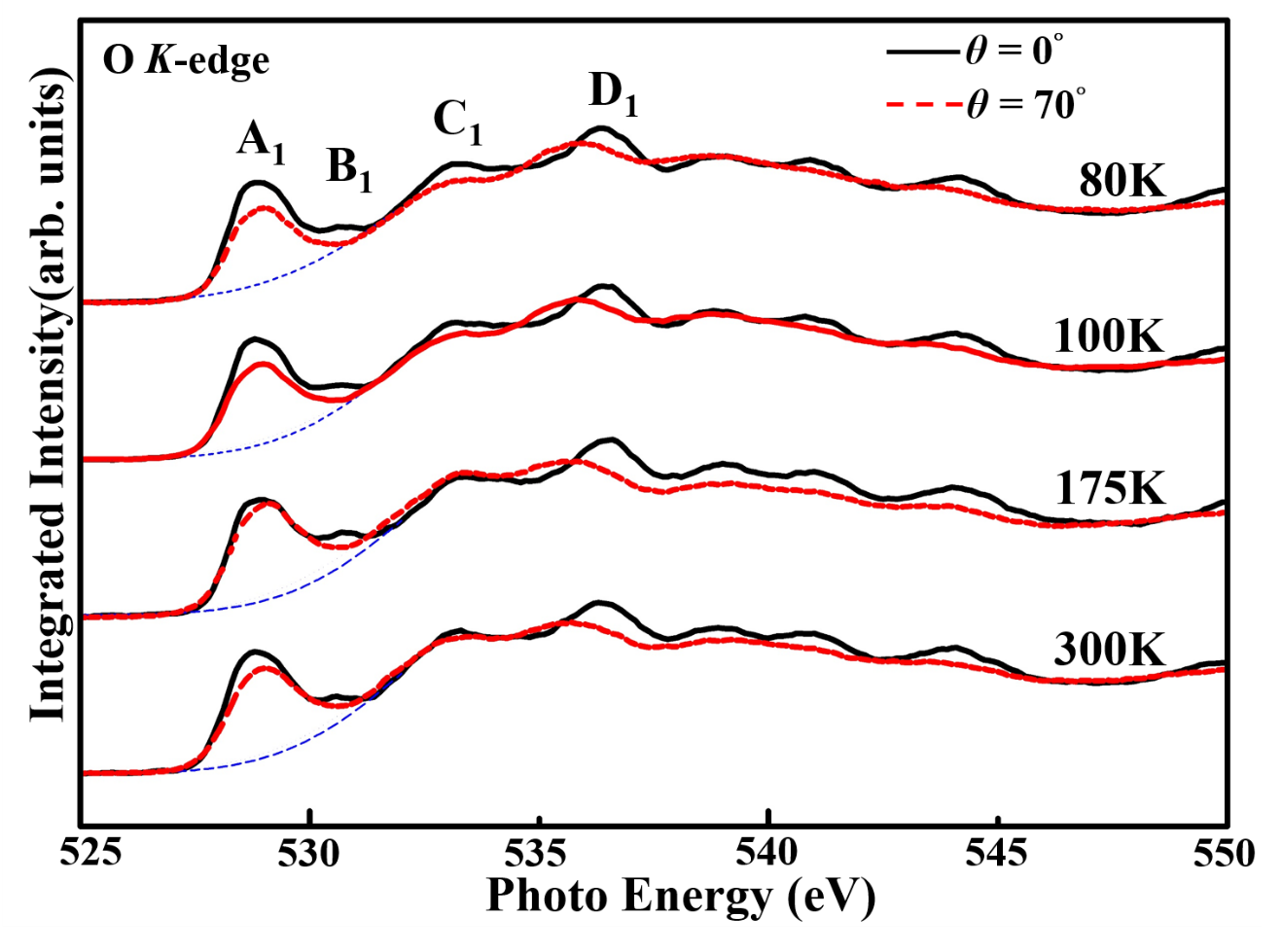


Fig. S1. Normalized O *K*-edge XANES spectra of YBCFO measured at 80, 100, 175 and 300 K.


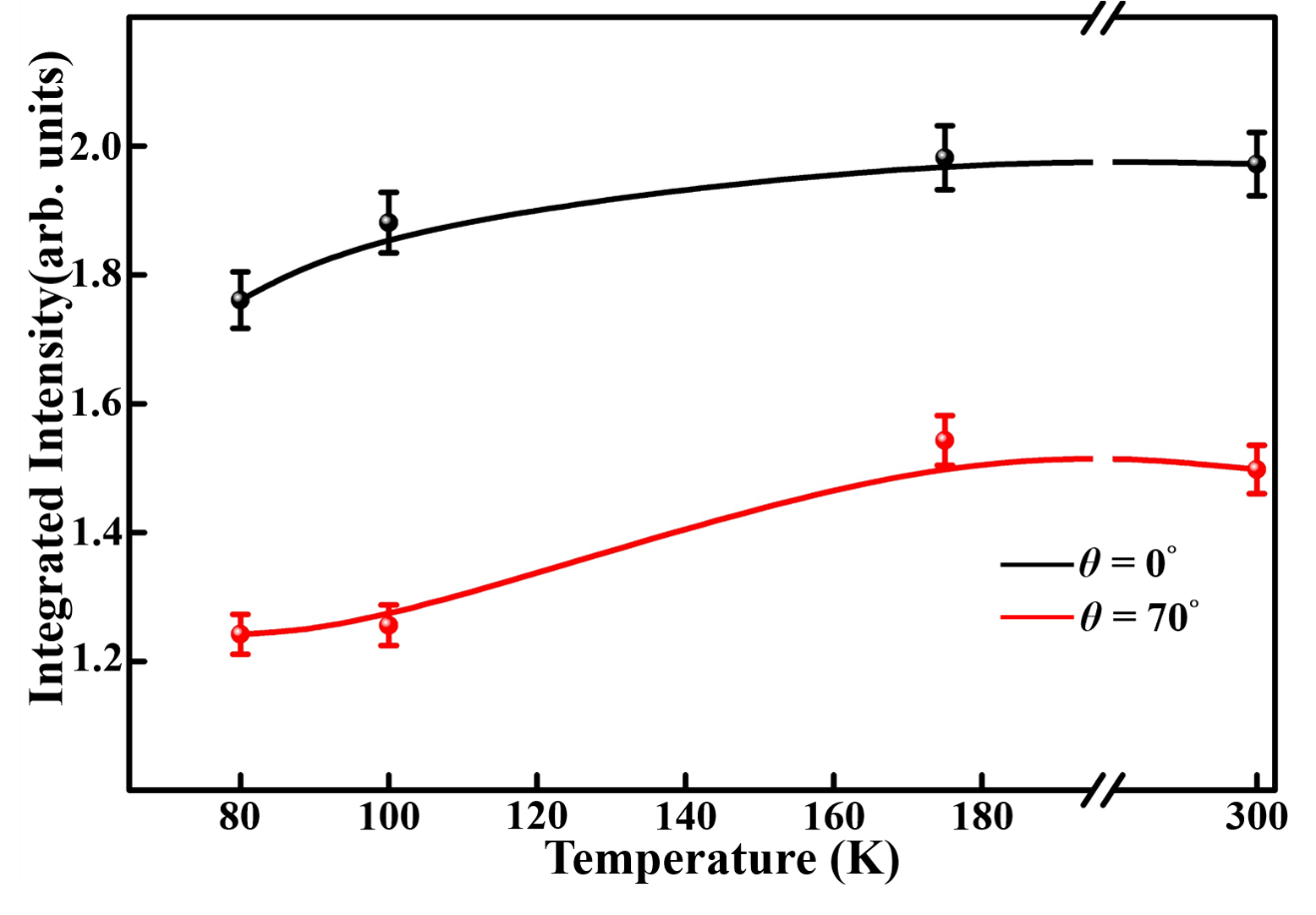


Fig. S2: Integrated O *K*-edge XANES intensity (between 526 to 532 eV in Fig. S1) at various temperatures in all spectra.

**References**

1. Groot, F. M. F. D. *et al.* Oxygen 1*s* X-ray-absorption edges of transition-metal oxides. *Phys. Rev. B* **40**, 5715-5723 (1989).

2. Asokan, K. *et al.* Effect of Co, Ni and Cu substitution on the electronic structure of hexagonal YMnO_3_ studied by x-ray absorption spectroscopy. *Appl. Phys. Lett.* **95**, 131901 (2009).

3. Chang, C. F. *et al.* Spin blockade, orbital occupation, and charge ordering in La_1.5_Sr_0.5_CoO_4_. *Phys. Rev. Lett.* **102**, 116401 (2009).

4. Braun, A. *et al.* High temperature oxygen near-edge X-ray absorption fine structure valence band spectra and conductivity of LaFe_3/4_Ni_1/4_O_3_ from 300 to 773 K. *Appl. Phys. Lett.* **99**, 202112 (2011).

5. Suntivich, J. *et al.* Estimating hybridization of transition metal and oxygen states in perovskites from O *K*-edge X-ray absorption spectroscopy. *J. Phys. Chem. C* **118**, 1856-1863 (2014).

6. Morin, M. *et al*. Incommensurate magnetic structure, Fe/Cu chemical disorder, and magnetic interactions in the high-temperature multiferroic YBaCuFeO_5_. *Phys. Rev. B* **91**, 064408 (2015).

7. Yoshikiyo, M., Yamada, K., Namai, A. & Ohkoshi, S.-I. Study of the electronic structure and magnetic properties of ε-Fe_2_O_3_ by first-principles calculation and molecular orbital calculations. *J. Phys. Chem. C* **116**, 8688-8691 (2012).

8. Tseng, Y. C. *et al*. Nonzero orbital moment in high coercivity ɛ-Fe_2_O_3_ and low-temperature collapse of the magnetocrystalline anisotropy. *Phys. Rev. B* **79**, 094404 (2009).

9. Lai, Y.-C. *et al.* Magnetic ordering and dielectric relaxation in the double perovskite YBaCuFeO5. *J. Phys.: Condens. Matter* 29, 145801 (2017).
